# Low-Loading of Pt Nanoparticles on 3D Carbon Foam Support for Highly Active and Stable Hydrogen Production

**DOI:** 10.3389/fchem.2018.00523

**Published:** 2018-11-06

**Authors:** Abdulsattar H. Ghanim, Jonathan G. Koonce, Bjorn Hasa, Alan M. Rassoolkhani, Wei Cheng, David W. Peate, Joun Lee, Syed Mubeen

**Affiliations:** ^1^Department of Chemical and Biochemical Engineering, University of Iowa, Iowa, IA, United States; ^2^Department of Chemical Engineering, University of Patras, Patras, Greece; ^3^Department of Earth and Environmental Sciences, University of Iowa, Iowa, IA, United States

**Keywords:** hydrogen evolution reaction, electrocatalyst, platinum nanoparticle, carbon foam, 3D support

## Abstract

Minimizing Pt loading is essential for designing cost-effective water electrolyzers and fuel cell systems. Recently, three-dimensional macroporous open-pore electroactive supports have been widely regarded as promising architectures to lower loading amounts of Pt because of its large surface area, easy electrolyte access to Pt sites, and superior gas diffusion properties to accelerate diffusion of H_2_ bubbles from the Pt surface. However, studies to date have mainly focused on Pt loading on Ni-based 3D open pore supports which are prone to corrosion in highly acidic and alkaline conditions. Here, we investigate electrodeposition of Pt nanoparticles in low-loading amounts on commercially available, inexpensive, 3D carbon foam (CF) support and benchmark their activity and stability for electrolytic hydrogen production. We first elucidate the effect of deposition potential on the Pt nanoparticle size, density and subsequently its coverage on 3D CF. Analysis of the Pt deposit using scanning electron microscopy images reveal that for a given deposition charge density, the particle density increases (with cubic power) and particle size decreases (linearly) with deposition overpotential. A deposition potential of −0.4 V vs. standard calomel electrode (SCE) provided the highest Pt nanoparticle coverage on 3D CF surface. Different loading amounts of Pt (0.0075–0.1 mg_Pt_/cm^2^) was then deposited on CF at −0.4 V vs. SCE and subsequently studied for its hydrogen evolution reaction (HER) activity in acidic 1M H_2_SO_4_ electrolyte. The Pt/CF catalyst with loading amounts as low as 0.06 mg_Pt_/cm^2^ (10-fold lower than state-of-the-art commercial electrodes) demonstrated a mass activity of 2.6 ampere per milligram Pt at 200 mV overpotential, nearly 6-fold greater than the commercial Pt/C catalyst tested under similar conditions. The 3D architectured electrode also demonstrated excellent stability, showing <7% loss in activity after 60 h of constant current water electrolysis at 100 mA/cm^2^.

## Introduction

Water electrolysis offers the potential to produce clean H_2_ sustainably from renewable electricity and water (Esposito et al., 2012; Chen et al., 2016; Jia et al., 2016). To design high-efficiency electrolyzers, highly active stable electrocatalysts are needed that can operate without corrosion in either strong acids or strong bases (Chen et al., 2018; Fu et al., 2018). To date, only electrocatalysts made of expensive platinum group metals (PGMs), specifically Pt, Ir, and Rh can deliver these needs (Angelo, 2007; Li et al., 2015; Zhang et al., 2015; Cheng et al., 2016; Tymoczko et al., 2016). Current practices to minimize mass loading of precious metals rely on constructing catalysts in the form of nanoparticles on low-cost 2D carbon supports (e.g., carbon cloth or carbon paper), with state-of-the-art commercial catalysts having a PGM mass loading of ~0.1–1 mg_PGM_/cm^2^ (Friedrich et al., 2004; Tymoczko et al., 2016; Park et al., 2018). However, for wide-spread practical applications, the target PGM loading level should be <0.125 mg_PGM_/cm^2^ (Benjamin et al., 2017). It is to be noted that considerable amount of revolutionary work has been dedicated to the development of earth-abundant catalysts for water electrolysis for some years (Cabán-Acevedo et al., 2015; Callejas et al., 2016; Liu et al., 2016; Tang et al., 2017). However, their activity and stability at present state are not sufficiently advanced to be performance-competitive (Faber et al., 2014).

**Figure 1:**
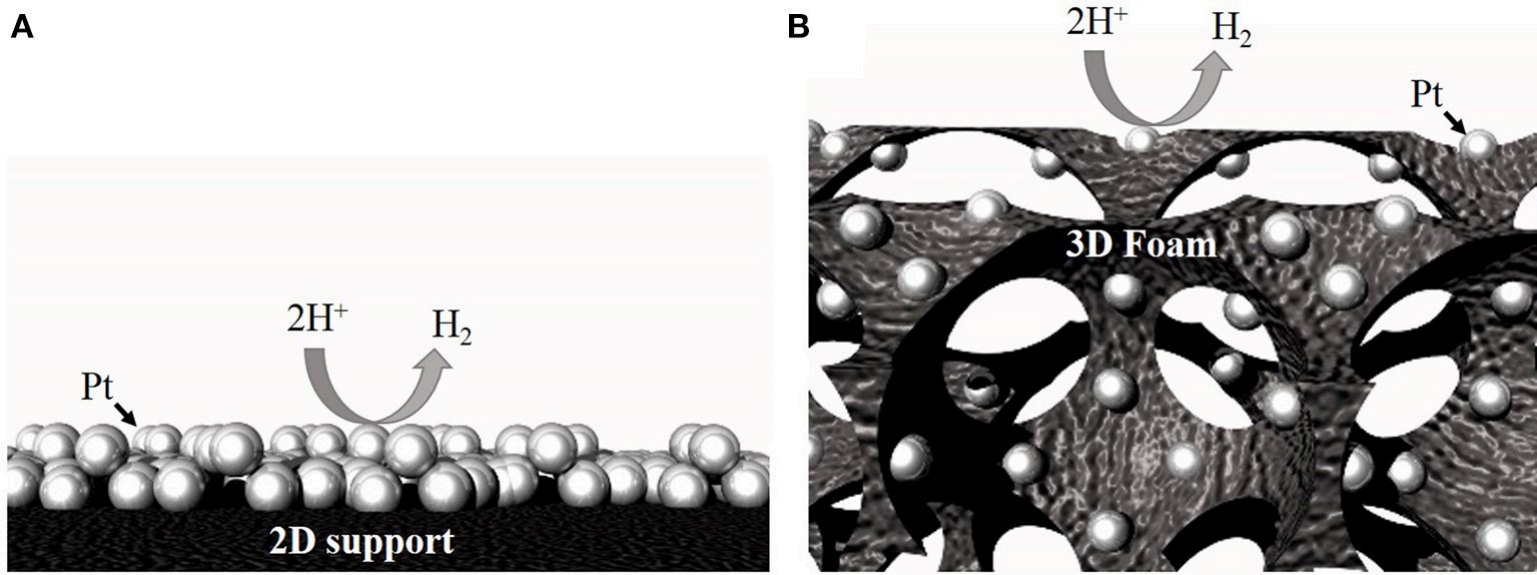
Schematic showing higher accessibility for the same number of particles on a 3D support **(B)** compared to a 2D support **(A)**.

Use of macroporous open-pore 3D catalyst supports (e.g., foam architecture; Figure 1) has been recommended to improve catalyst utilization efficiency and lower the PGM metal loadings (Li et al., 2015; Pierozynski and Mikolajczyk, 2016). These foams have pore sizes ranging from 0.2 to 5 mm and can serve as both current collectors and as supports to load catalysts (Xing et al., 2011; Huang et al., 2017). Compared to traditional 2D supports, 3D open-pore foam architecture provides large surface area, enhanced electrolyte penetration, and gas diffusion, excellent structural integrity, and fast 3D electron transfer pathways (Friedrich et al., 2004; Aldalbahi et al., 2018). Additionally, the continuous, open-pore architecture of the 3D foams, is expected to provide excellent mass and ion-transport to and from the catalyst site, significantly improving the bubble convection when operated at high water electrolysis current densities. Recently, several approaches have been demonstrated to load PGMs on 3D open-pore metallic foams. For example, van Drunen et al. (2014) deposited platinum nanoparticles electrolessly on Ni foam and evaluated its performance for different electrocatalytic reactions. Li et al. (2015) demonstrated successful deposition of complete-monolayer of Pt on a 3D nickel foam substrate using Au or Ag buffer layer for hydrogen evolution reaction.

While many possible 3D foams are amenable to loading electrocatalyst, for large-scale cost-effective production of catalyst/support assemblies, the support should be made of low-cost materials and should be highly resistant to chemical and electrochemical corrosion. In this regard, carbon foam supports are of particular interest due to their low-cost and excellent chemical stability. In fact, 3D carbon foams (CF) have been used successfully as current collectors for microbatteries (Johns et al., 2011), metal ion removal (Friedrich et al., 2004), supercapacitors (Fischer et al., 1997), and enzymatic fuel cells (Kizling et al., 2017). However, to the best of our knowledge, no report exists on optimizing PGM loading on 3D carbon foams and benchmarking its electrocatalytic activity for important reactions such as hydrogen evolution reaction (HER).

In the present work, we synthesize uniformly dispersed Pt nanoparticles on inexpensive 3D open-pore carbon foam support using electrodeposition and optimize its catalytic activity for HER reaction. We elucidate the dependence of Pt nanoparticle diameter, density, and mass loading on electrodeposition potential and deposition charge density. The Pt/CF catalyst with loading as low as 0.06 mg/cm^2^ (~10-fold lower than state-of-the-art commercial electrodes) yield the highest mass and surface area specific activity that is six and 67 times the activity of commercial Pt/C catalyst tested under similar conditions. Moreover, the Pt/CF catalyst also demonstrate highly stable HER activity in acidic conditions after a continuous long-term chronopotentiometry run at high current density (@100 mA/cm^2^).

## Materials and methods

### Materials

Reticulated vitreous carbon (RVC) foam substrates with 100 pores per inch (PPI) and a specific surface area of 2 × 10^3^ square feet per cubic foot, and 1.96 × 10^−2^ ohms sq^−1^ electrical surface resistance were purchased from McMaster-Carr (Duocell RVC Foam, ERG Aerospace). Commercial Pt/C cloth (0.5 mg/cm^2^, 67 m^2^/g_Pt_, 20 wt%, E-TEK; Muthuswamy et al., 2013) and bare carbon cloth were purchased from Fuel Cell Store. All chemicals were purchased from Fisher Scientific (sodium chloride (NaCl, 99%), potassium hydroxide (KOH, 86.3%), sulfuric acid (H_2_SO_4_, 96.3%), hydrochloric acid (HCl, 36.5%), nitric acid (HNO_3_, 69%), potassium tetrachloroplatinate (K_2_PtCl_4_, 99.9%), and copper sulfate pentahydrate (CuSO_4_•5H_2_O, 98%). The standard solutions for Pt and Rh detection using ICP-MS (1,000 ppm platinum and 1,000 ppm rhenium) were purchased from Inorganic Ventures. All solutions for deposition and catalyst testing were prepared using ultrapure water (18 MΩ/cm).

### Pt nanoparticle deposition on carbon foam surface

A room temperature three-electrode electrochemical set-up was used for Pt electrodeposition with carbon foam as the working electrode, Pt wire as the counter electrode and a Saturated Calomel Electrode (SCE) as the reference electrode. The plating solution was made of 0.5 M sodium chloride and 3 mM potassium tetrachloroplatinate (K_2_PtCl_4_) with pH adjusted to 4 using dilute HCl. All depositions were carried out on a new carbon foam substrate with dimensions cut to 2 × 1 × 0.3 cm. The Pt nanoparticles were deposition following a potentiostatic double pulse deposition technique (Figure S1). In this technique, each pulse consisted of a constant potential deposition period (till 15 mC of charge was passed) and a constant potential surface activation period (5 s). The target deposition potentials were chosen from linear sweep voltammetry curves (Figure S2). The target deposition charge density was obtained by controlling the total number of pulses. Four different deposition potentials [-0.2,−0.3,−0.4, and−0.5 V vs. SCE (V_SCE_)] and four different deposition charge densities (50, 100, 150, and 300 mC/cm^2^) were investigated for this study. A potential of 0 V_SCE_ was chosen as the surface activation potential for all depositions to remove any adsorbed chloride and hydrogen atoms from the deposited Pt surface.

### Characterization

Scanning Electron Microscopy (SEM) was done by using a field emission SEM (Hitachi S-4800), and the particles size and density were determined by using Image-J software. For each sample, three separate areas were analyzed with Image-J to give an average for the particles diameter and density. A low-magnification SEM image of carbon foam loaded with Pt nanoparticles is shown in Figure S3.

Mass loading of Pt was determined using inductively coupled plasma mass spectrometry (ICP-MS). One square centimeter area of the carbon foam with deposited Pt particles was digested in 1 mL of aqua regia. The aqua regia was prepared from concentrated hydrochloric acid and concentrated nitric acid that was combined in a molar ratio of 3:1 HCl:HNO_3_. This was diluted by a factor of 10,000 to bring the concentration to the ppb range. Samples were analyzed on a Thermo X-series II ICP-MS instrument. All samples were doped with a known amount of Re to correct for instrumental drift. Multiple Pt isotopes were measured to verify that polyatomic interferences were not an issue with these samples. Calibration curves were made for each isotope (^194^Pt, ^195^Pt, ^196^Pt) (Figure S4). All resulted in consistent concentrations, and the final values were calculated from a weighted average using the natural abundances of the three major platinum isotopes.

Electrochemical active surface area (ECSA) of Pt nanoparticles on carbon foam was calculated using copper underpotential deposition method (Cu-UPD) technique. The Pt loaded carbon foam served as the working electrode, Pt wire served as the counter electrode and Ag/AgCl as the reference electrode. The Cu-UPD experiments were carried out in 0.001 M CuSO_4_in 0.5 M H_2_SO_4_ solution. The electrodes were cycled between the potential limits of 0.15–0.8 vs. V_Ag/AgCl_ at a scan rate of 20 mV/s until the charges associated with Cu stripping remained constant for each cycle (Figure S5). By comparing this value to the charge of the formed Cu monolayer on polycrystalline Pt (410 μC/cm^2^), and by knowing the total mass loading of Pt, the ECSA can be calculated using the following equation:

$$\begin{array}{l} ECSA_{Pt}\;\left(\frac{cm^{2}}{mg_{Pt}}\right)=\;\left[\frac{Q_{Cu-UPD\;\left(\mu C\right)}}{410\frac{\mu C}{cm^{2}}\;X\;mg_{Pt}\;}\right] \end{array}$$

### Hydrogen evolution reaction experiments

Platinum nanoparticle-loaded carbon foam samples were tested for hydrogen evolution reaction (HER) in acid (1 M H_2_SO_4_) and alkaline (1 M KOH) electrolyte. A three-electrode electrochemical set-up was used for all HER studies. The carbon foam with Pt loading was used as the working electrode, pure platinum wire as the counter electrode, Ag/AgCl as a reference electrode for the acidic electrolyte, and Hg/HgO as the reference electrode for the alkaline electrolyte. Cyclic voltammetry (CV) runs were done at a scan rate of 100 mV/s. For all representative CV curves shown in this study, the electrodes were cycled for at least 30+ times (totaling 50–70 C/cm^2^) which allowed for a stable and repeatable curve. The overpotential values were calculated from the final cycle of the CV graphs. All CV runs were compensated for iR losses. For stability tests, a constant cathodic current density of 100 mA/cm^2^ was applied and the potential was monitored as a function of time (chronopotentiometry technique). Similar strategy of cycling the electrodes as explained above was practiced prior to all stability tests.

## Results and discussion

### Effect of deposition potential on Pt nanoparticle size and density

**Figure 2:**
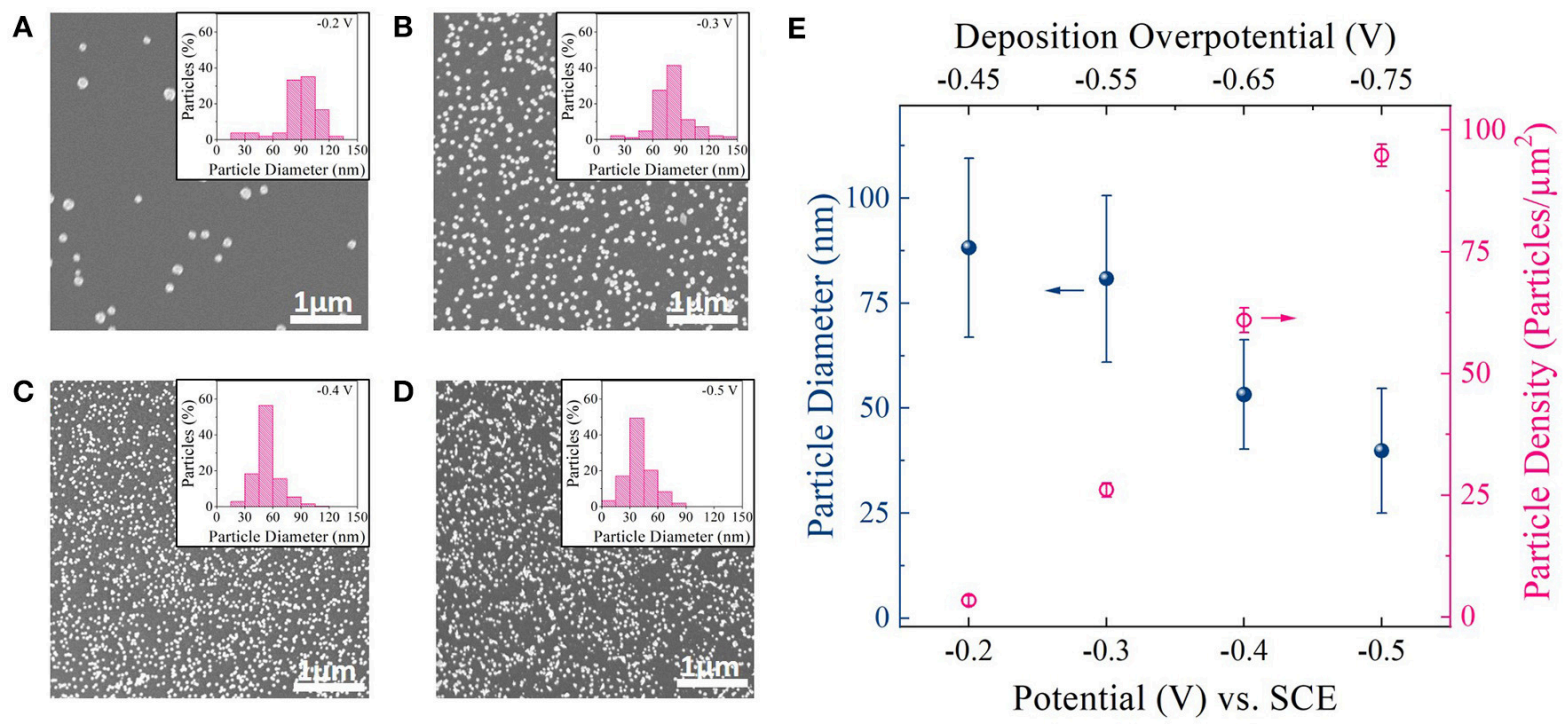
**(A–D)** SEM images of Pt particles on carbon foam supports deposited at−0.2 V_SCE_,−0.3 V_SCE_, −0.4 V_SCE_, and −0.5 V_SCE_. Inset: Histogram showing particle size distribution for each deposition potential. **(E)** Average diameter and density of Pt nanoparticles deposited at different deposition potential pulses. Top x-axis: Deposition overpotential (η) value for Pt deposition.

Figures 2A–D shows representative top-down scanning electron microscopy (SEM) images of Pt nanoparticles deposited on carbon foam for different deposition potential pulses. Four different deposition potentials,−0.2,−0.3,−0.4, and−0.5 V vs. Standard Calomel Electrode (V_SCE_) (from linear sweep voltammetry; Figure S2) were chosen for this study. As shown in Figure S2, these deposition potentials are well negative to the equilibrium potential for Pt deposition (+0.25 V_SCE_) (Liu et al., 2012). Deposition potentials more negative than−0.5 V_SCE_ resulted in hydrogen evolution (${\text{E}}_{\text{H}+/\text{H}2,\text{pH}=4}^{0}$ = −0.48 V_SCE_) during Pt deposition and consequently was not chosen. All depositions were carried for an equivalent deposition charge density of 150 mC/cm^2^. Corresponding i-t deposition transients for each deposition potential is shown in Figure S6.

The SEM images (Figures 2A–D) revealed well dispersed Pt nanoparticles on the carbon surface for all deposition potentials. Figure 2E shows a plot of particle diameter and density as a function of deposition potential as measured from ImageJ software. Corresponding histogram plots are shown in the inset of SEM images. From the SEM images, histogram plots, and Figure 2E one could observe a clear decrease in particle size and increase in particle density as the deposition overpotential increased. This suggests that at low electrodeposition overpotential (−0.2 V_SCE_), the nucleation rate is slower than the rate of the nucleus growth on the carbon foam surface (Hussain et al., 2017). That is, it is more favorable for Pt to deposit on the existing nuclei and grow before the next nucleus is formed on carbon surface (heterogeneous reduction), resulting in larger and fewer number of Pt nanoparticles (Figure 2A). With the increase in deposition overpotentials (−0.3, −0.4, and −0.5 V_SCE_), the rate of nucleation increases resulting in densely distributed smaller Pt nanoparticles (Figures 2B–D). In fact both particle diameter and particle density follows classical heterogeneous nucleation and growth mechanism (Pei et al., 2017), with particle diameter decreasing linearly with deposition overpotential and particle density increasing to the cube power with increasing deposition overpotential (Figure S7).

Since for electrocatalysis application, high surface coverage of smaller Pt nanoparticles on a given support surface directly translates to high geometric activity, we chose to work with higher deposition overpotentials for further catalyst optimization studies. Particularly −0.4 V_SCE_ was chosen as our optimal deposition potential, since it provided the highest Pt surface area to support surface area among the deposition potentials chosen for this study (Figure S8).

### Effect of deposition charge on Pt mass loading

To address the challenging cost targets associated with Pt, it is very important to significantly reduce Pt mass loading without compromising its geometric and specific activity. Mass loading of Pt on CF was controlled by simply tuning the deposition time (mass deposited is directly proportional to the charge passed). Four different charge densities were chosen to electrodeposit Pt on CF surface (50, 100, 150, and 300 mC/cm^2^; denoted hereafter as Pt/CF_50_, Pt/CF_100_, Pt/CF_150_, and Pt/CF_300_). These deposition charge densities were selected such that the Pt mass loading for the highest deposition charge (Pt/CF_300_) was lower than the recommended target Pt loading level of <0.125 mg_Pt_/cm^2^ for electrocatalytic applications. As discussed above, a deposition potential pulse of −0.4 V_SCE_ was selected for all these studies.

Table 1 column 2 summarizes Pt mass loading (mg_Pt_/cm^2^) results measured using inductively coupled plasma mass spectrometry (ICP-MS; see Materials and Methods section) for different deposition charge densities. As expected, the Pt mass loading increased with increasing deposition charge density, varying from 0.0075 mg_Pt_/cm^2^ for Pt/CF_50_ to 0.1015 mg_Pt_/cm^2^ for Pt/CF_300_ sample.

**Table 1:** Tabulated data of each platinum loading level using a 1 cm^2^ geometric area of Pt/CF.

**Sample**	**Mass Pt (mg_Pt_/cm^2^)**	**Electrochemical active surface area**		**Pt surface area (${\text{cm}}_{\text{Pt}}^{2}$)**
		**(${\text{m}}_{\text{Pt}}^{2}$/g_Pt_)**	**(${\text{cm}}_{\text{Pt}}^{2}$/mg_Pt_)**	
Pt/CF_50_	0.0075	11.066	110.66	0.83
Pt/CF_100_	0.0325	5.877	58.77	1.91
Pt/CF_150_	0.059	6.322	63.22	3.73
Pt/CF_300_	0.1015	6.732	67.32	6.80

**Figure 3:**
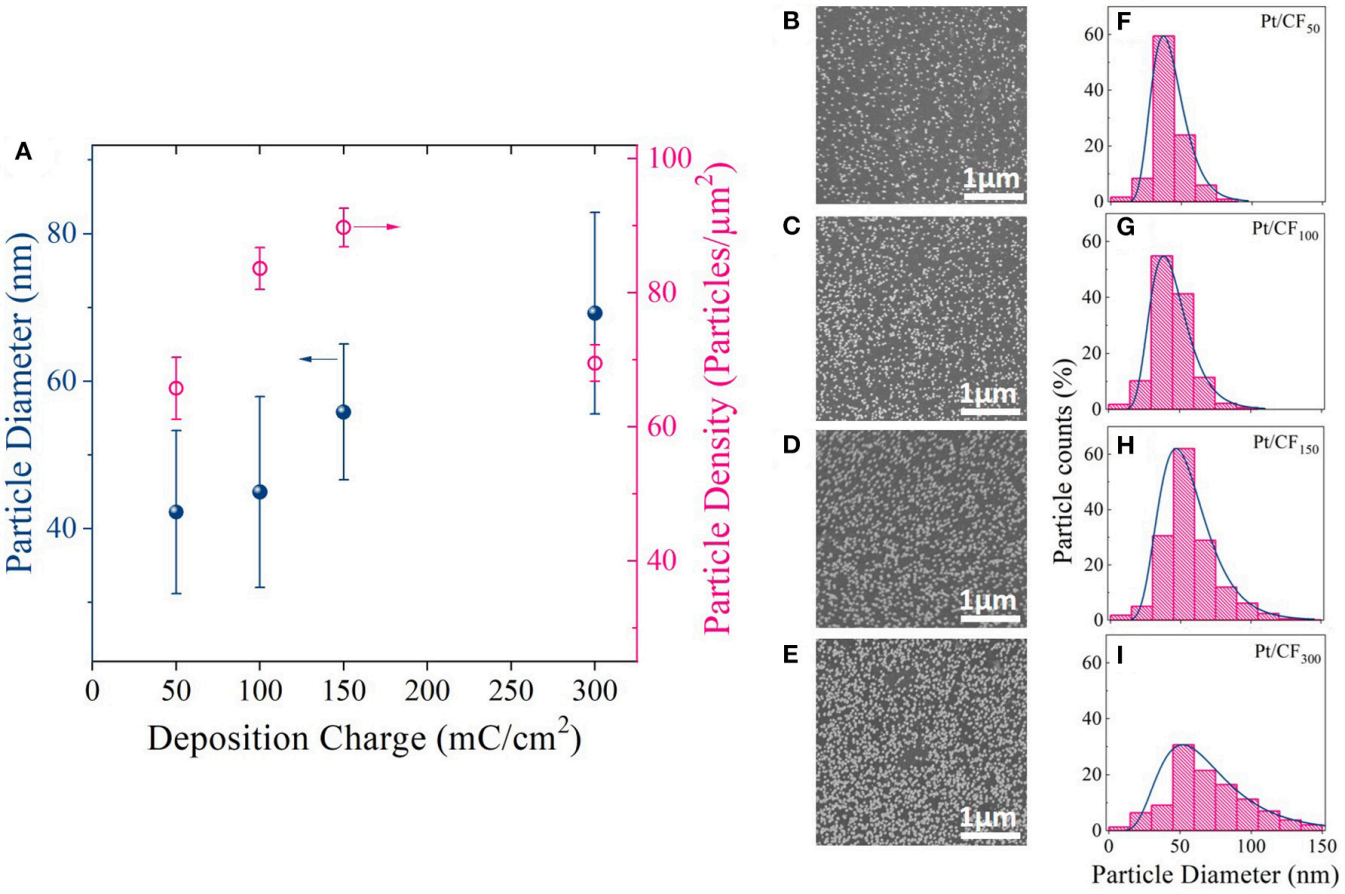
**(A)** Average Pt particle diameter and density for different deposition charge densities (50, 100, 150, and 300 mC/cm^2^). The deposition potential was kept constant at −0.4 V_SCE_. **(B–E)** SEM images and **(F–I)** corresponding histogram plots showing the particle diameter distribution for each charge density.

Figures 3B–E shows SEM images of Pt deposition progression as a function of deposition charge density. Figure 3A is a plot of particle density and diameter as a function of mass loading. For Pt/CF_50_, the particle density on average was 66 particles/μm^2^ (Figure 3A red trace), and the particles remained sparsely distributed on the carbon foam surface. Doubling the deposition charge density increased the particle density by ~27% to 84 particles/μm^2^ while effectively keeping the average particle diameter constant (43 nm; Figure 3A black blue trace). This observed increase in particle density with deposition charge signifies that the Pt deposition on 3D foam follows a progressive nucleation and growth process. That is, as deposition time increases, more and more Pt nuclei are formed at the carbon surface, increasing the surface area of Pt per unit volume of the catalyst support. This is consistent with the trend observed for measured surface area (${\text{cm}}_{\text{Pt}}^{2}$) of the catalyst which shows an increase with increasing deposition charge density (Table 1 Column 4). For an instantaneous nucleation and growth mechanism, the particle density should remain relatively constant as the deposition charge increases (Grujicic and Pesic, 2002).

The histogram plots for the particle diameter (Figures 3F–I) further elucidates the Pt deposition mechanism with increasing deposition charge. A log-normal distribution was observed for each loading level. A log-normal distribution is expected when nucleation dominates growth, which increases the number of small particles with time with simultaneous growth of larger particles deposited initially. The above observation is very similar to what was observed by Teran et al. (2010) who calculated the grain size distribution for a random nucleation and growth process. However, with further increase in mass loading (Pt/CF_300_) the particle density starts to decrease (Figure 3A, red trace), possibly due to coalescence of the neighboring particles as evidenced by the increase in average particle diameter from 43 to 70 nm (Figure 3A, blue trace). The coalescence of the particles observed for Pt/CF_300_ is also evident from the histogram plot Figure 3H, where the peak of the distribution shifts to the right with a larger spread.

### HER activity

**Figure 4:**
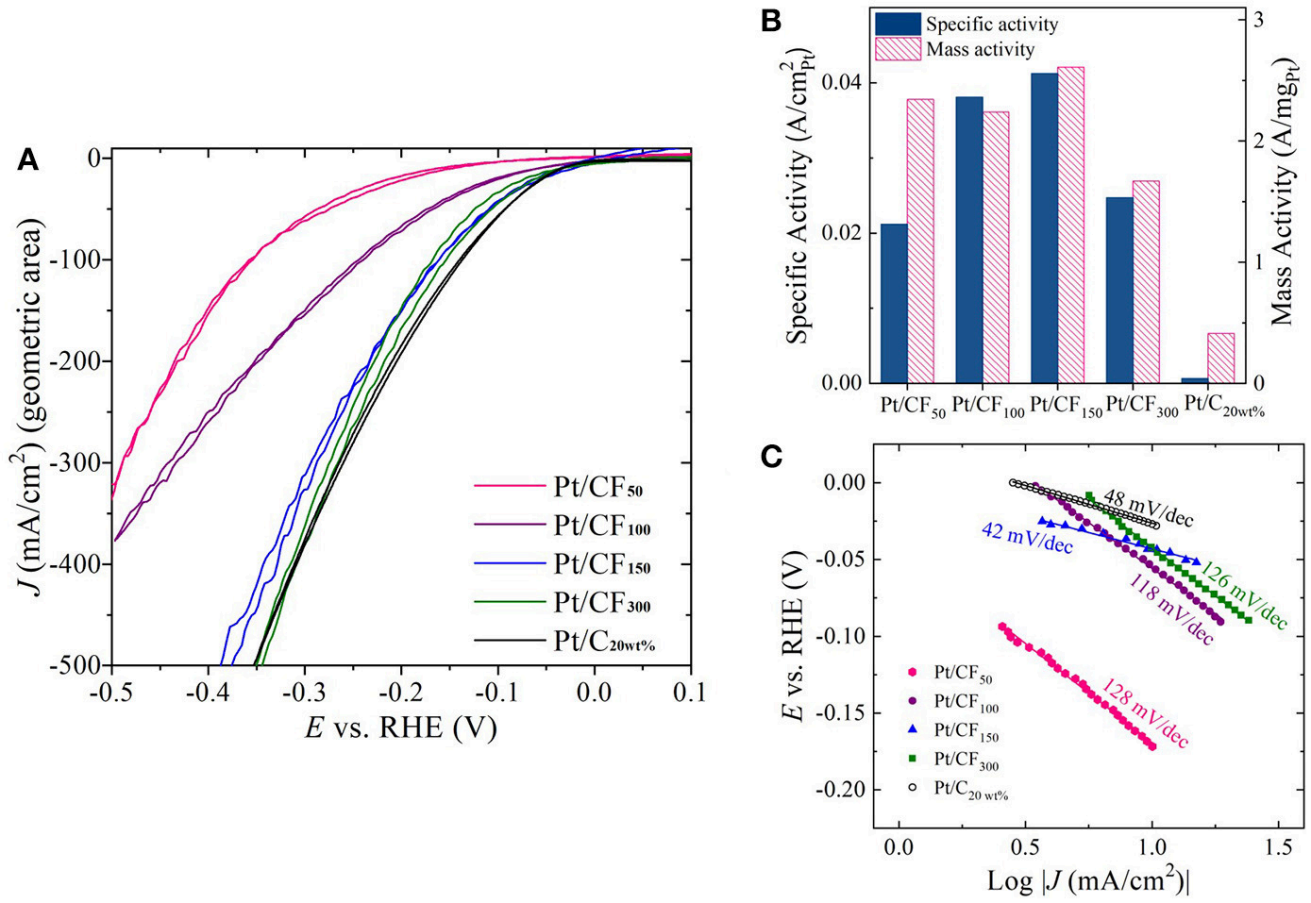
**(A)** Cyclic voltammetry (CV) sweeps showing HER activity in 1 M H_2_SO_4_ for four Pt loading levels (50, 100, 150, and 300 mC/cm^2^) as well as commercial Pt-loaded carbon cloth. **(B)** Plot of specific activity and mass activity of each catalyst at η = 200 mV. **(C)** Tafel slope plots constructed for each Pt loading level.

The effect of Pt mass loading on HER activity in acidic and alkaline conditions was evaluated using a standard three-electrode electrochemical cell. Figure 4A shows geometric HER current densities (mA/${\text{cm}}_{\text{geo}}^{2}$) obtained in 1 M H_2_SO_4_ for Pt/CF samples by sweeping voltages from 0.1 V vs. reversible hydrogen electrode (V_RHE_) to −0.5 V_RHE_ at a rate of 100 mV/s (see the Materials and Methods section for more details). All Pt/CF electrodeposited samples were directly used as working electrodes for the HER tests. Commercial Pt/carbon cloth with 20 wt. % Pt and mass loading of 0.5 mg_Pt_/cm^2^ (denoted hereafter as Pt/C_20wt%_) was also selected as a reference point and studied under the same conditions. For all measurements, the HER currents were measured as a function of ohmic-corrected potential. As shown in Figure 4A, the Pt/CF catalysts demonstrated increasing HER currents with increase in Pt mass loading. Particularly, Pt/CF_150_ and Pt/CF_300_ catalyst showed excellent HER activity, as evidenced by the very small overpotential (η) of 370 and 340 mV needed to deliver a high current density of 500 mA/cm^2^. Figure S10 provides cyclic voltammograms from the 10th CV cycle for each Pt loading with its corresponding Tafel slope analysis. These values are comparable to state-of-the-art commercial 2D Pt/C_20wt%_ sample (Figure 4A, black trace). It should be noted that the Pt/CF_150_ and Pt/CF_300_ samples had Pt mass loading 10- and 5-fold lower than the commercial Pt/C_20wt%_electrode and HER results were obtained under quiescent electrode/electrolyte conditions (no vigorous stirring and/or electrolyte flow).

Although geometric current density serves as a practical metric to compare catalyst performance, for optimal catalyst design it is important to compare mass activity (A/mg_Pt_) and specific activity (A/${\text{cm}}_{\text{Pt}}^{2}$) as a function of Pt loading. Figure 4B, red bars show mass activity measured at η = 200 mV for different deposition charge densities. The mass activity of Pt/CF samples remained more or less independent to initial deposition charge density, with the highest mass activity of 2.61 A/mg_Pt_ obtained for Pt/CF_150_, which is 6.3 times higher than that of the state-of-the-art commercial Pt/C_20wt%_ (0.41 A/mg_Pt_). The mass activity decreased with further Pt mass loading (Pt/CF_300_; 1.67 A/mg_Pt_), possibly due to the reduction in Pt mass utilization due to coalescence of smaller particles as discussed above (Figures 3E,I). However, we note that the lowest mass activity obtained for Pt/CF_300_ (1.67 A/mg_Pt_) is still four times higher than that of the commercial catalyst.

The specific activity (A/${\text{cm}}_{\text{Pt}}^{2}$) for each catalyst was determined from its mass activity (A/mg_Pt_) and electrochemical active surface area (ECSA; ${\text{cm}}_{\text{Pt}}^{2}$/mg_Pt_). The measured ECSA for different mass loading (in ${\text{cm}}_{\text{Pt}}^{2}$/mg_Pt_ and ${\text{m}}_{\text{Pt}}^{2}$/g_Pt)_ is summarized in Table 1 Column 3. Cu-underpotential (Cu-UPD) deposition was used to determine the ECSA assuming a specific charge of 420 μC/${\text{cm}}_{\text{Pt}}^{2}$ per monolayer Cu deposited on the Pt surface (see Materials and Methods Section). No Cu-UPD was observed on bare carbon foam substrate. The specific activity of the catalyst increased with Pt loading with Pt/CF_150_ catalyst showing nearly 67 times greater specific activity compared to commercial Pt/C_20wt%_ catalyst (Figure 4B, blue bars). The specific activity decreased with further increase in deposition charge density (Pt/CF_300_) likely due to decreased Pt mass utilization caused by nanostructure/morphology changes as discussed above. The Tafel analysis (Figure 4C) gave Tafel slope values of 128, 118, 42, and 126 mV/dec for Pt/CF_50_, Pt/CF_100_, Pt/CF_150_, and Pt/CF_300_ when plotted for current density ranges 3–30 mA/cm^2^. The Tafel slope for Pt/CF_150_ was lower than commercial Pt/C_20wt%_ (48 mV/dec), which is consistent with this catalyst having the highest mass and specific activity. When tested in alkali media (1M KOH), the Pt/CF catalysts exhibited similar trends to that observed in acidic electrolyte (Figures S9, S11). The Pt/CF_150_ catalyst exhibited the best catalytic performance with a specific activity of 0.126 A/${\text{cm}}_{\text{Pt}}^{2}$ (@η = 400 mV) with a reasonably small Tafel slope (105 mV/dec) for alkaline conditions. XRD Data for different Pt loading levels is provided in Figures S12, S13 provides current density-volage profile for Pt/CF_150_ sample with Graphite as the counter electrode.

**Figure 5:**
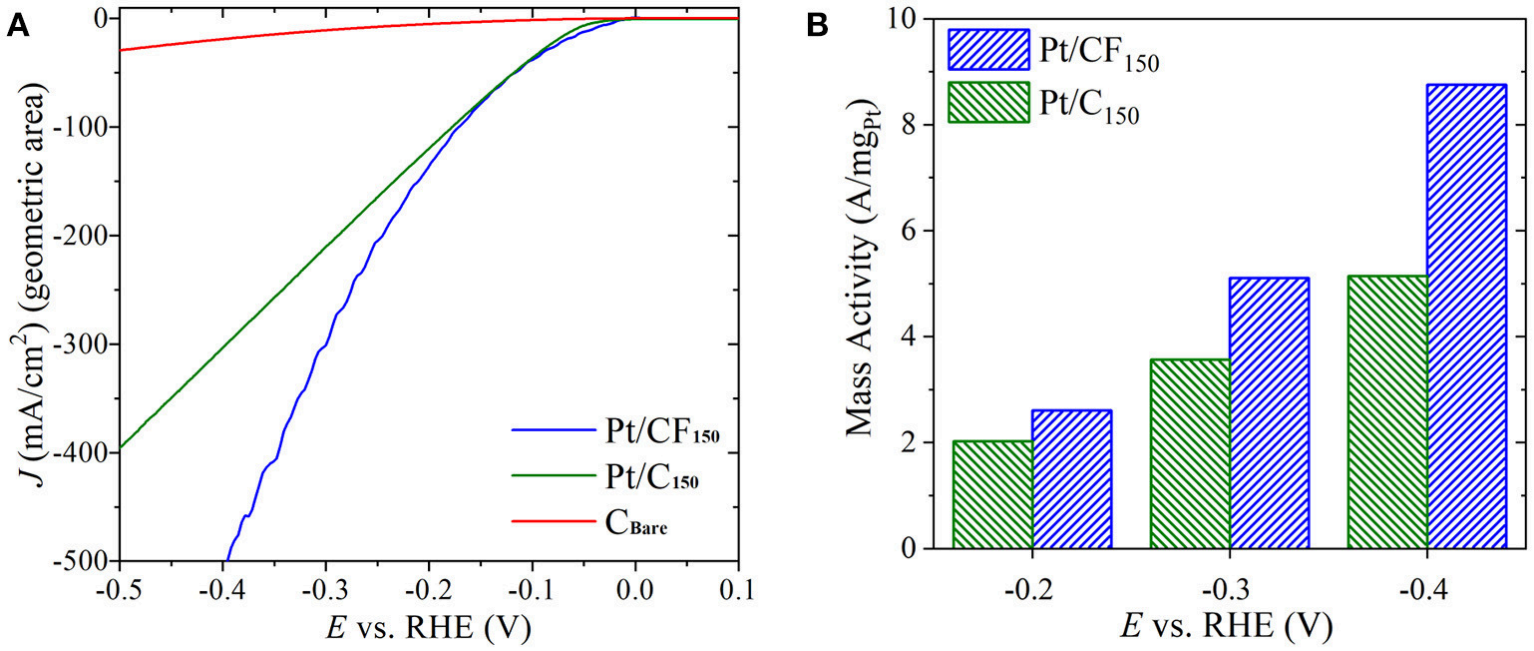
**(A)** Forward scan from cyclic voltammetry (CV) sweeps showing HER activity in 1 M H_2_SO_4_ for Pt loaded on 3D carbon foam with 150 mC/cm^2^ loading (Pt/CF_150_), Pt loaded on carbon cloth with 150 mC/cm^2^ loading (Pt/C_150_), and bare carbon cloth (C_Bare_). The potential is iR-compensated. **(B)** Mass activity of Pt/CF_150_ and Pt/C_150_ at three overpotentials.

To better understand the role of 3D carbon foam substrates on observed HER enhancement, electrochemical tests were carried out on 2D carbon cloth with Pt mass loading similar to the best performing Pt/CF electrode (Pt/CF_150_). The Pt was loaded on the 2D carbon cloth following the exact electrodeposition protocols. The amount of Pt deposited was controlled by tuning the deposition charge and HER tests were carried out using the same electrochemical set-up with similar reactor volume and electrode positioning. As shown in Figure 5A, for the same mass loading, the Pt/C and Pt/CF electrode (represented as Pt/C_150_ and Pt/CF_150_) have similar overpotential values up to current densities of 75 mA/cm^2^ (see Figure S14 for Tafel slope analysis). However, with increasing current densities, the Pt/CF_150_ behaved superior compared to its 2D counterpart_._ For example, to achieve 400 mA/cm^2^, the Pt/CF_150_ required a modest overpotential of 350 mV compared to 500 mV for Pt/C_150_ electrode. No significant HER currents were observed on bare carbon cloth (C_Bare_). The observed superior performance for Pt/CF_150_ at higher current densities further corroborates that 3D CF substrate provides better bubble convection and access to catalyst sites compared to 2D carbon cloth. We also normalized the performance of both Pt/C_150_ and Pt/CF_150_ electrode by their corresponding actual catalyst loadings and found that the difference in mass activity between Pt/CF_150_ and Pt/C_150_ increases with increasing HER overpotential (Figure 5B). At an overpotential of 400 mV (−0.4V_RHE_), the mass activity of Pt/CF was 8.75 A/mg_Pt_, almost 70% higher than that of Pt/C electrode.

### Long-term stability

**Figure 6:**
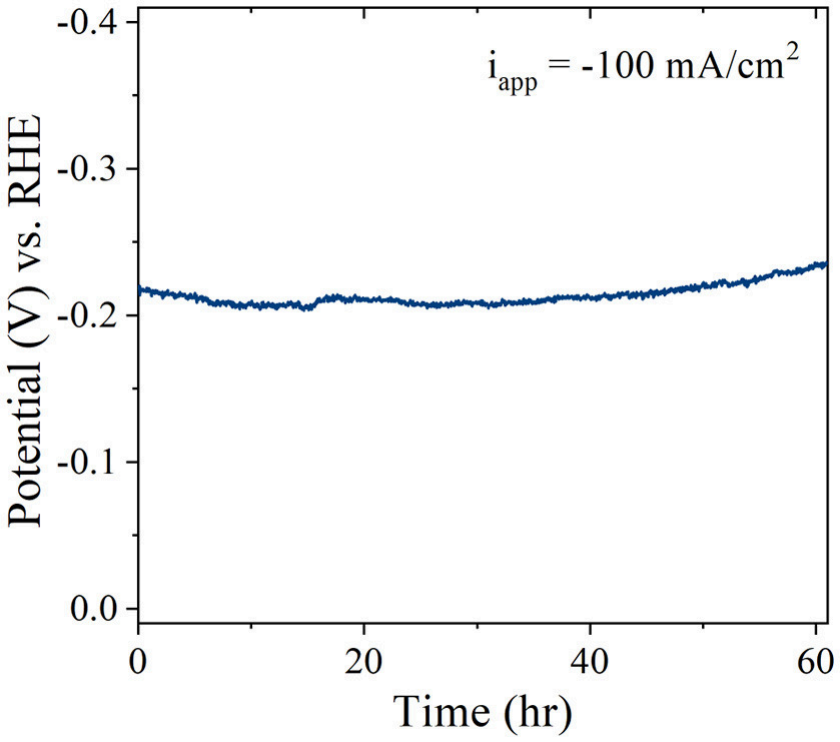
Stability of Pt/CF_150_ operating at−100 mA/cm^2^ in 1 M H_2_SO4.

To evaluate the long-term stability of the Pt/CF catalysts, accelerated degradation studies were performed for the best performing Pt/CF_150_ catalyst in acidic conditions. The stability was assessed by monitoring the increase in overpotential in 1 M H_2_SO_4_ for an applied current density of 100 mA/cm^2^. No *iR* compensation was carried out for the stability runs. Figure 6 shows that after 60 hours of continuous operation the overpotential increased by less than 16 mV (7%) in acid. It is to be noted that the current density used here for stability measurement is 10-fold higher than that of the most reported literature values for low PGM loaded materials (Li et al., 2015). The catalysts after stability testing were characterized by SEM images, which showed no obvious change to the structural morphology (particle diameter and density), indicating that the Pt nanoparticles are extremely stable and bound strongly to the carbon foam support even under high current density operation.

In conclusion, we demonstrated a simple solution-processed electrodeposition technique to load low amounts of Pt nanoparticles on low-cost, high surface area 3D carbon foam support for HER reaction. We established the dependence of particle diameter and density as a function of deposition potential and charge density and investigated the HER activity of the Pt-loaded carbon foams. All synthesized Pt/CF catalysts exhibited excellent mass activities that are superior to the state-of-the-art commercial Pt/C catalyst. For the best performing Pt/CF catalyst, the mass and specific activity were 6.3 and 67 times higher than commercial Pt/C catalysts. Also, the best performing catalyst showed excellent stability with minimal degradation when operated at high current densities. We attribute this significant improvement in catalytic activity observed for Pt/CF samples to the following reasons. (i) The electrodeposition technique allows binder-free deposition of Pt directly on the electroactive site of the support facilitating efficient charge transport across support/catalyst interface. (ii) Enhanced mass transport: the 3D open-pore foam architecture provides enhanced electrolyte penetration and improved diffusion of H^+^ ions and H_2_ to and from the Pt nanoparticle surface. (iii) Increased surface area of the 3D carbon foam providing easy access to the catalytic sites.

## Author contributions

AG and JK designed and carried out all the synthesis, characterization, and data analysis. BH performed Tafel analysis. AR, WC, and JL assisted AG and JK with material synthesis and microscopy characterization. DP performed ICPMS measurements. SM supervised the work, designed experiments with AG and JK. All authors were involved in manuscript writing.

### Conflict of interest statement

The authors declare that the research was conducted in the absence of any commercial or financial relationships that could be construed as a potential conflict of interest.
