# Supplementary material for: Low-Loading of Pt Nanoparticles on 3D Carbon Foam Support for Highly Active and Stable Hydrogen Production
Source: Front Chem. 2018 Nov 6;6:523. doi: 10.3389/fchem.2018.00523 (PMC6232265; doi:10.3389/fchem.2018.00523)
Supplement: Supplementary file 1 [file Table_1.DOCX]

Supporting Information For:

**Low-loading of Pt nanoparticles on 3D carbon foam support for highly active and stable** **hydrogen production**

**Abdulsattar H. Ghanim,^‡#^ Jonathan G. Koonce,^‡#^ Bjorn Hasa,^⊥^ Alan M. Rassoolkhani,^#^ Wei Cheng,^#^ David W. Peate^†^, Joun Lee,^#^ and Syed Mubeen^*#^**

^#^Department of Chemical and Biochemical Engineering, University of Iowa, IA, 52242

^⊥^Department of Chemical Engineering, University of Patras, Patras, Greece, 26504

^†^Department of Earth & Environmental Sciences, University of Iowa, IA, 52242

***Correspondence:**Syed Mubeen
syed-mubeen@uiowa.edu

**Author Contributions**

‡A. H. Ghanim and J. G. Koonce contributed equally to this work.


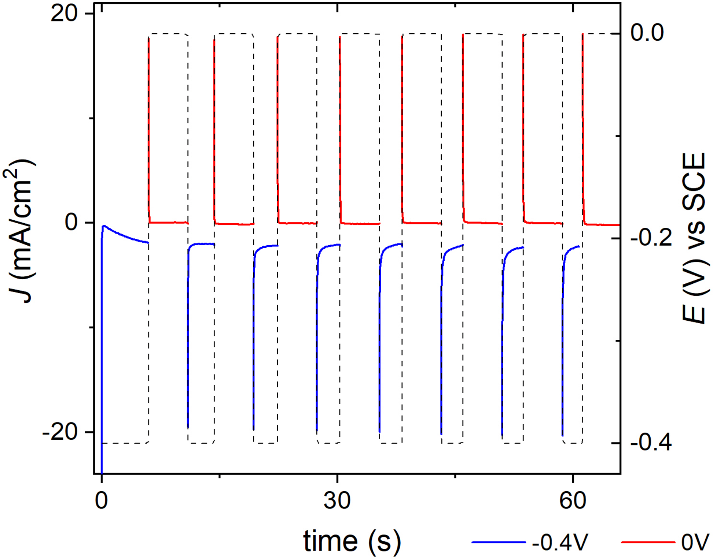


**Figure S1.** Representative pulsed-potential deposition profile. The blue line shows current density at the applied potential of -0.4 V_SCE_ and the red line at 0 V_SCE_. Black dotted line shows the applied potential.


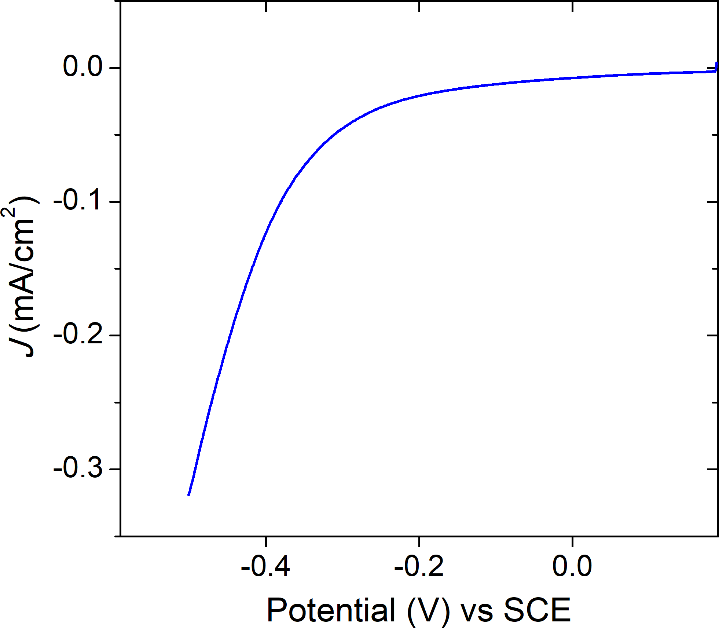


**Figure S2.** LSV of Pt deposition on RVC substrate using 0.5 M NaCl and 3 mM K_2_PtCl_4_ electrolyte

**Figure S3.** Low magnification SEM image of 3D open-pore carbon foam substrate. (Inset) High magnification SEM image shows the detail of Pt nanoparticles on the surface of the carbon foam substrate.

**
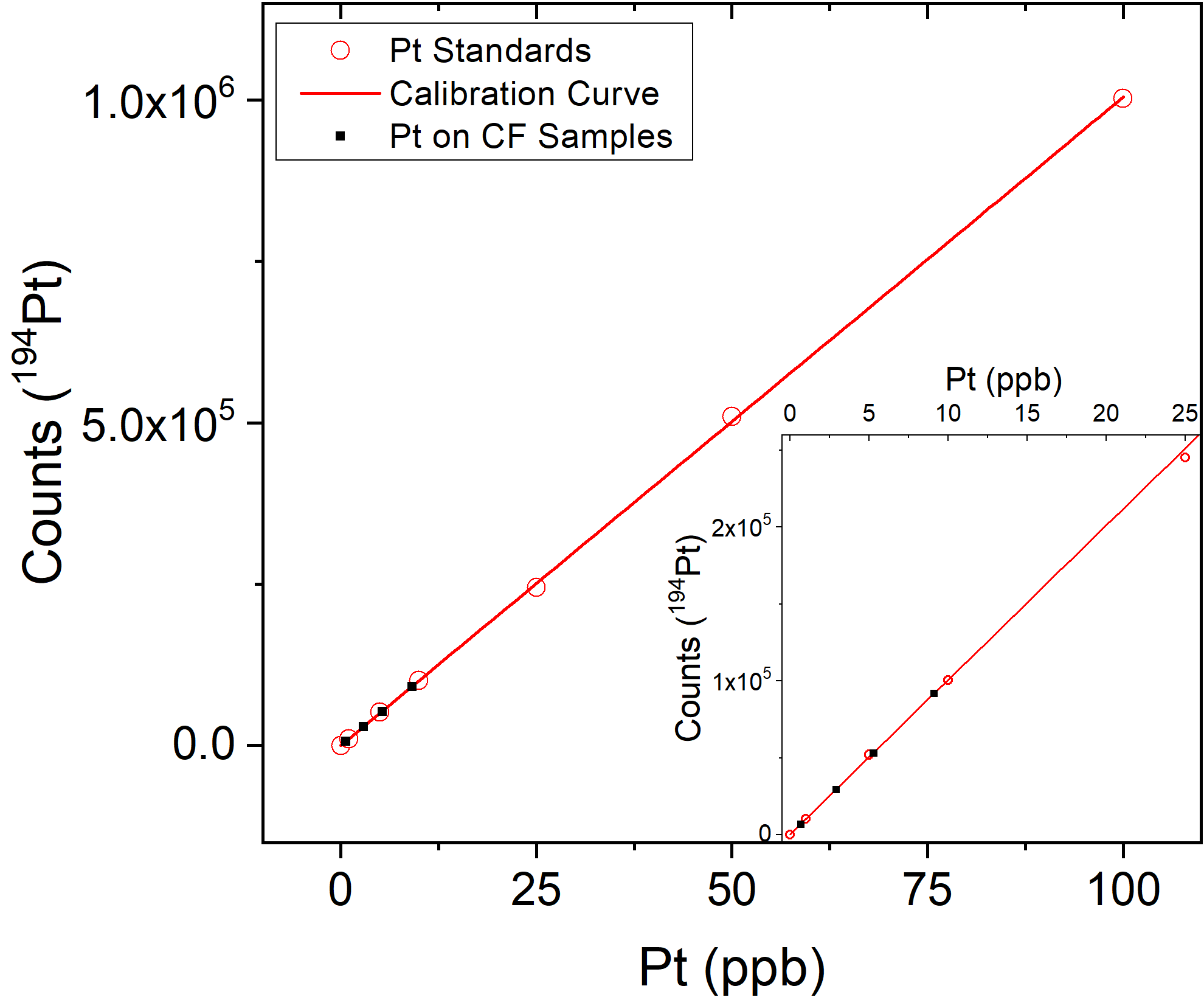
**

**Figure S4.** Calibration curve for ^194^Pt showing the calibration standards and the measured values for the four Pt loading levels. Inset shows detail of the lower concentration range that the Pt on CF samples were in. Similar calibration curves were made for ^195^Pt and ^196^Pt which gave consistent concentrations for all four samples.


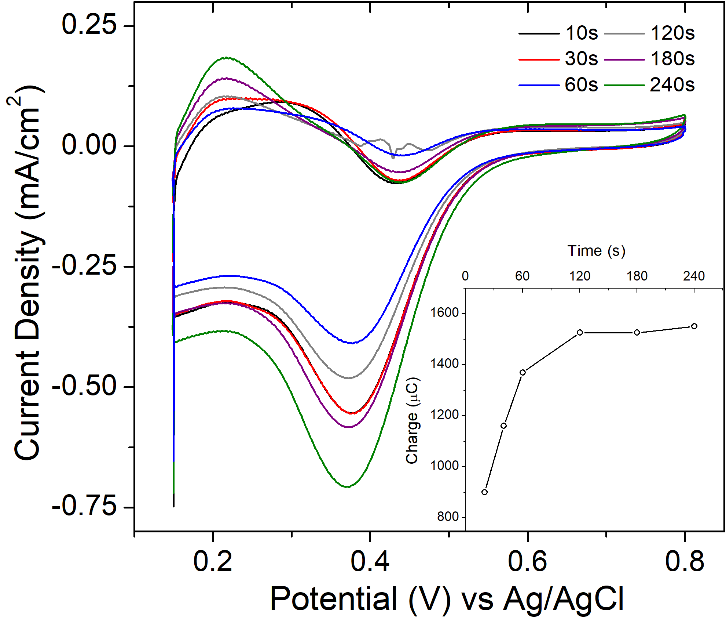


**Figure S5.** Cyclic voltammogram of underpotential copper deposition and stripping on a 150 mC/cm^2^ Pt/CF sample. The inset shows the deposition time vs stripping charge. The charge remains constant after two minutes, indicating complete monolayer coverage of Cu on Pt. The same calculations were done for all Pt loading levels.


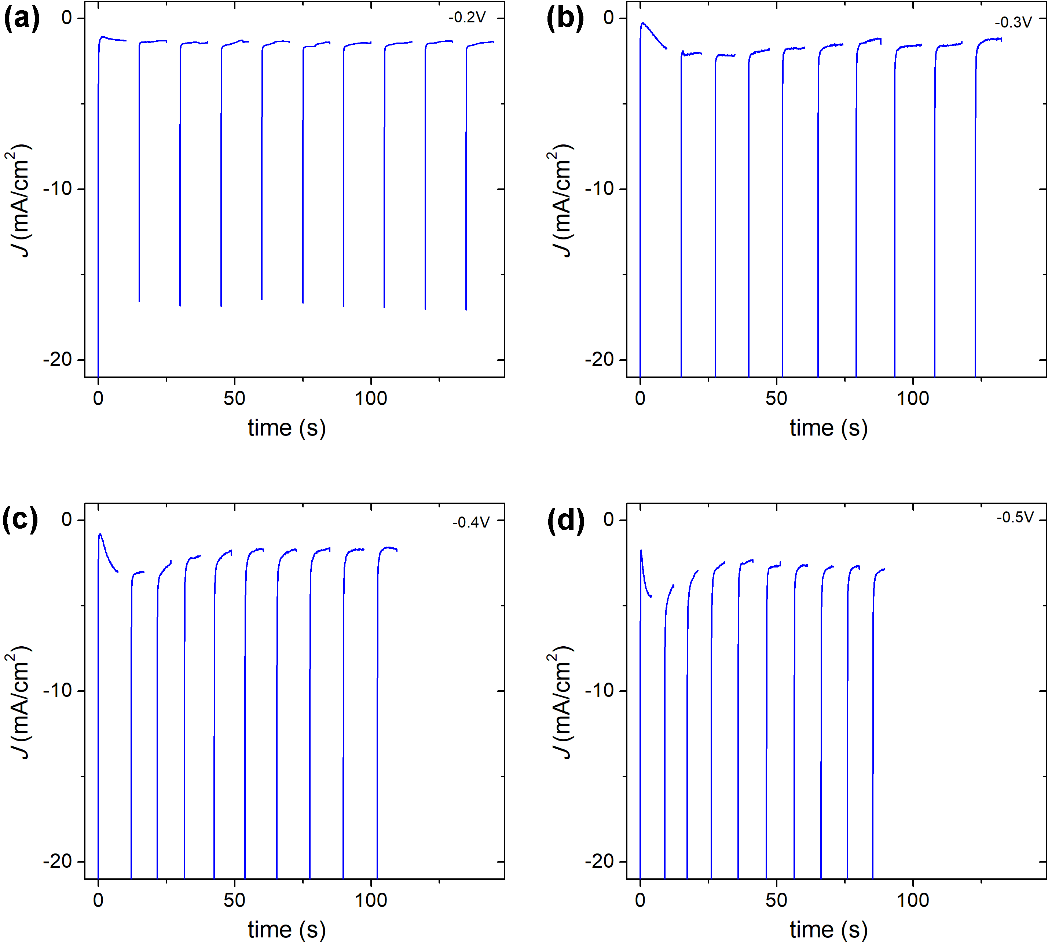


**Figure S6.** Deposition profiles for (a) -0.2 V (b) -0.3 V (c) -0.4 V and (d) -0.5 V vs. SCE. Each profile shows a total deposition charge of 150 mC/cm^2^. With increasing negative overpotential, the total time for each deposition decreases as the rate that the same amount of charge passes increases.

**(b)**

**(a)**


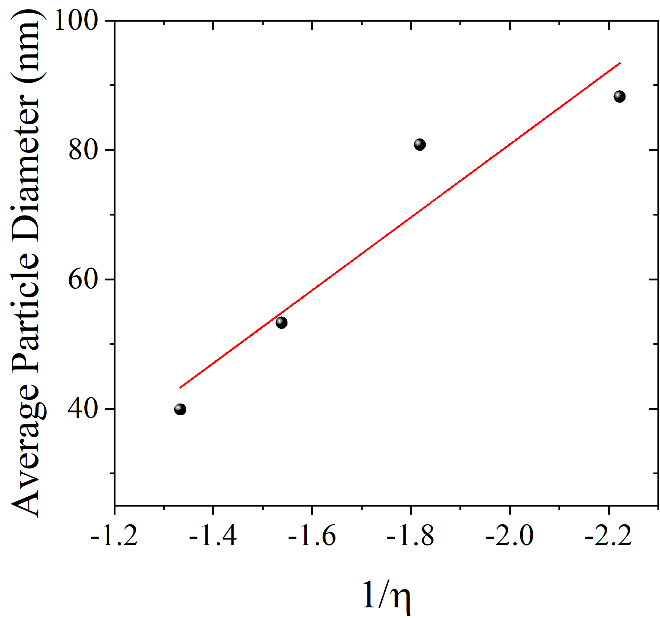

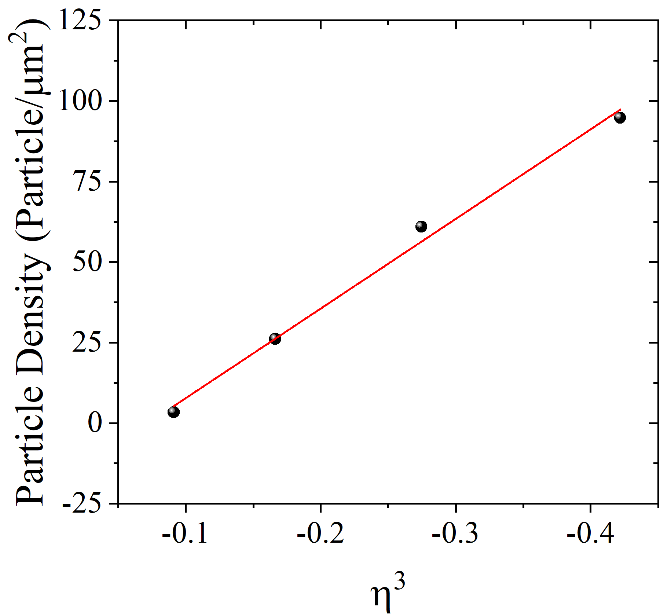


**Figure S7**. (a) Average particle diameter plotted versus inverse deposition overpotential. (b) Particle density versus the cube of overpotential.


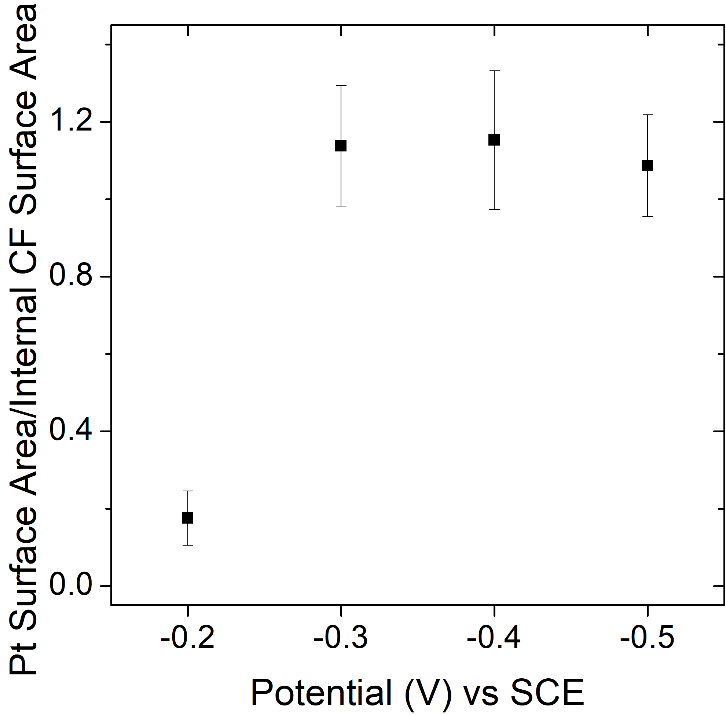


**Figure S8**. A plot of Pt surface area over internal CF surface area.


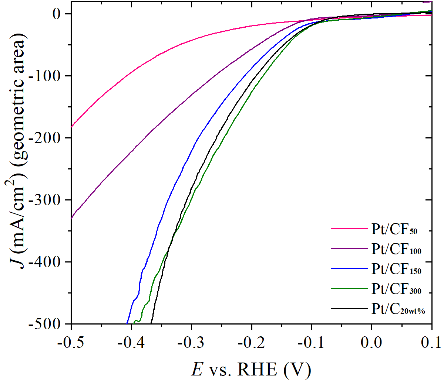

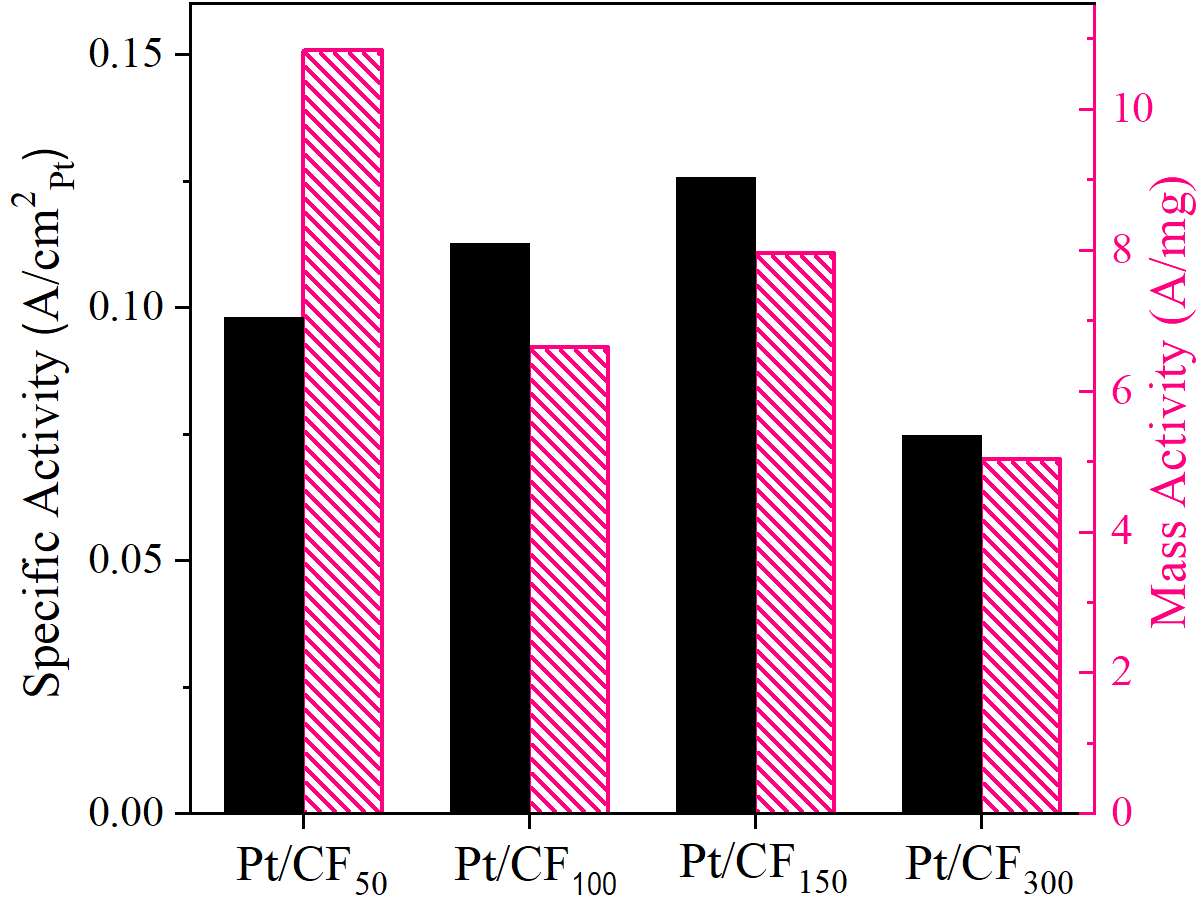

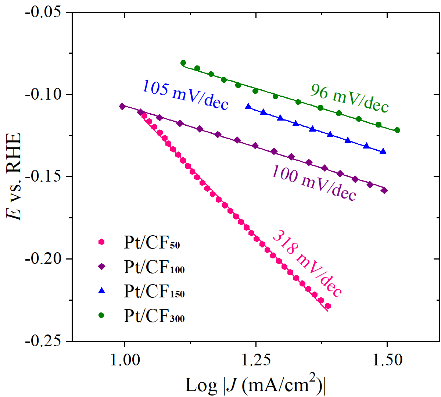


**(c)**

**(b)**

**(a)**

**Figure S9.** (a) Forward scan from cyclic voltammetry (CV) sweeps showing HER activity in 1 M KOH for each of four Pt loading levels (50, 100, 150, and 300 mC/cm^2^) and Pt/C_20wt%_. The currents are capacitive-corrected and the potential is iR-compensated. (b) Specific activity and mass activity of each sample taken at an overpotential of η = 400 mV. These current densities have been corrected for capacitive current. (c) Tafel slope plots constructed for each Pt loading level.




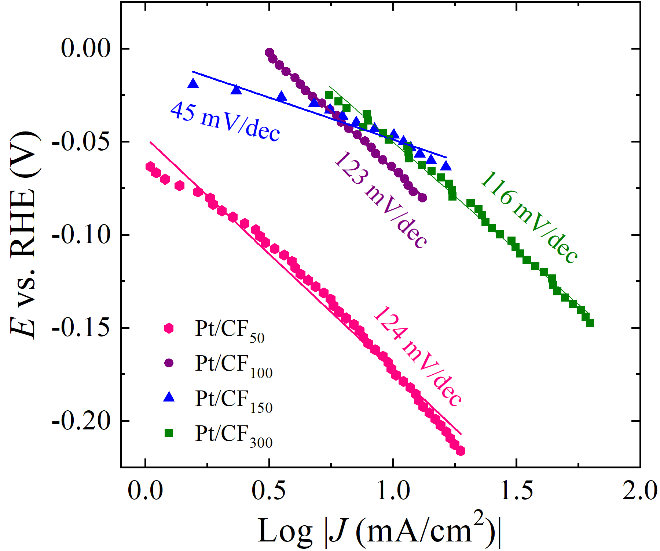


**(a)**

**(b)**

**Figure S10.** Cyclic voltammograms from the 10^th^ CV cycle for each Pt loading level in 1 M H_2_SO_4_


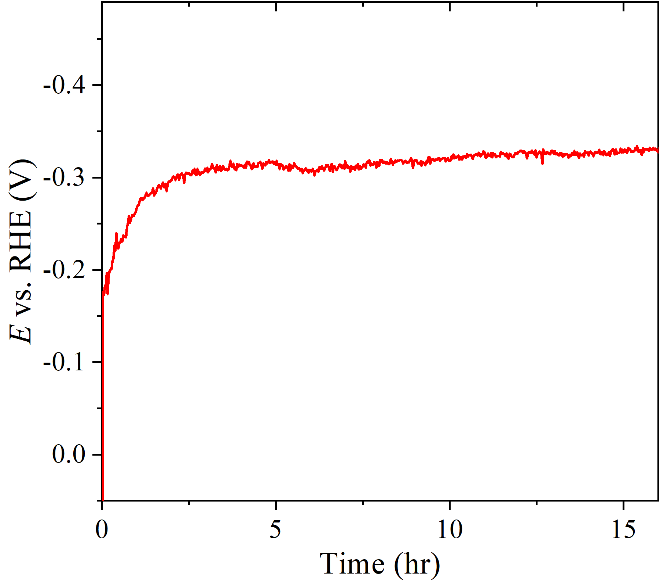


**Figure S11.** Stability of Pt/CF_150_ operating at ‑100 mA/cm^2^ in 1 M KOH.


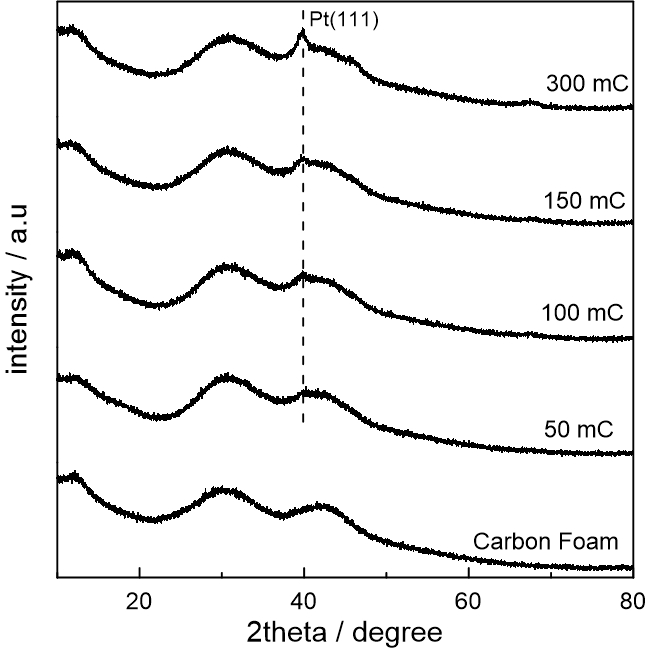


**Figure S12.** XRD data for the 50, 100, 150, and 300 mC Pt loading levels as well as for bare carbon foam. The Pt(111) peak is weakly visible in the highest loading levels.





**Figure S13.** Forward scan from cyclic voltammetry (CV) in 1 M H_2_SO_4_ showing similar performance after 30 cycles with a Pt CE and a graphite CE.


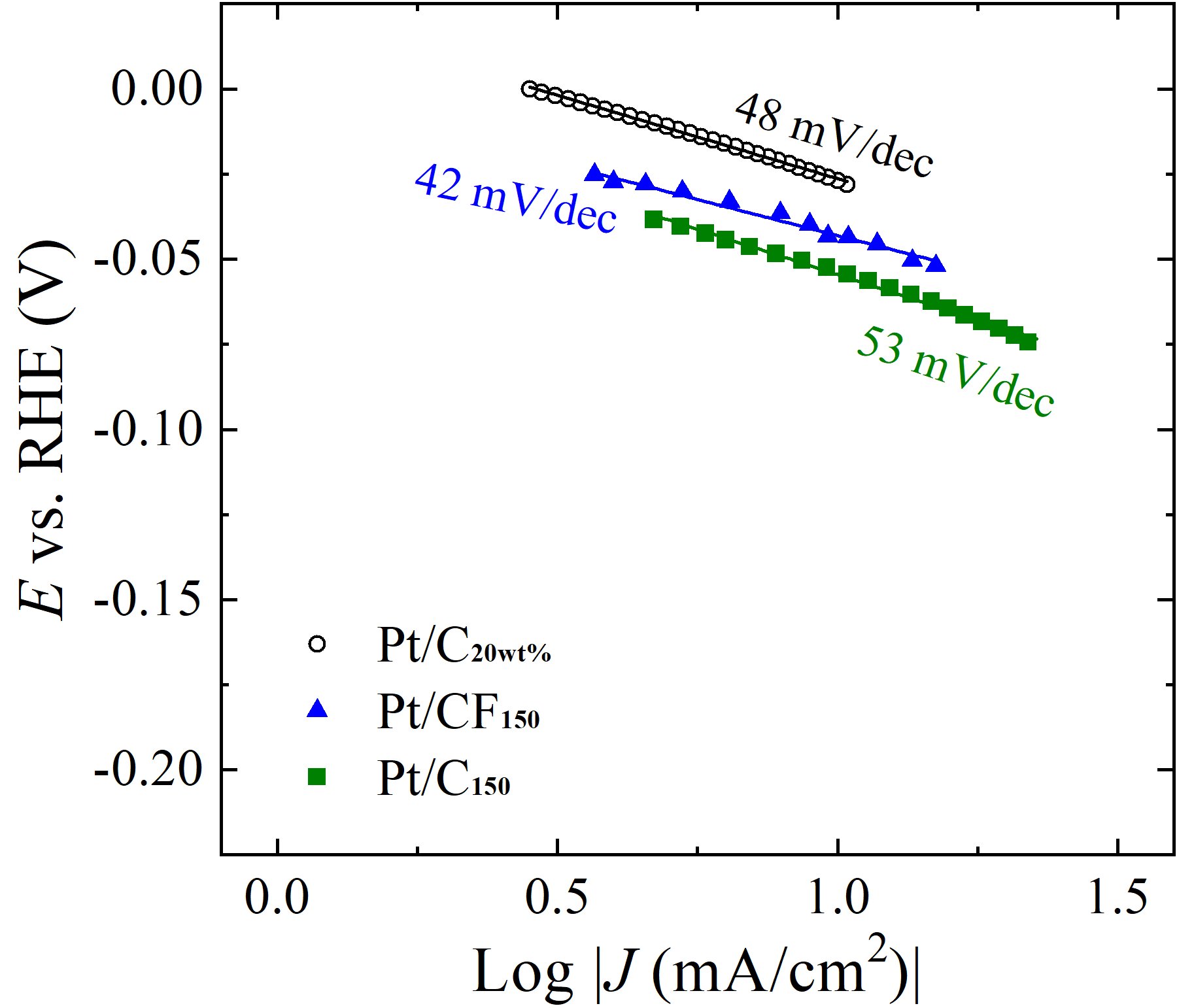


**Figure S14.** Tafel slope plots constructed from HER activity in 1 M H_2_SO_4_ for commercially purchased Pt-loaded carbon cloth (Pt/C_20wt%_), Pt loaded on 3D carbon foam with 150 mC/cm^2^ loading (Pt/CF_150_), and Pt loaded on carbon cloth with 150 mC/cm^2^ loading (Pt/C_150_).
